# Effect of Metformin on Short-Term High-Fat Diet-Induced Weight Gain and Anxiety-Like Behavior and the Gut Microbiota

**DOI:** 10.3389/fendo.2019.00704

**Published:** 2019-10-18

**Authors:** Shuqin Ji, Lingwei Wang, Lei Li

**Affiliations:** ^1^Guangdong Provincial Key Laboratory of Brain Connectome and Behavior, The Brain Cognition and Brain Disease Institute (BCBDI), Shenzhen Institutes of Advanced Technology, Chinese Academy of Sciences, Shenzhen, China; ^2^Shenzhen Key Laboratory of Respiratory Diseases, Shenzhen Key Laboratory of Pathogenic Microorganisms and Bacterial Resistance, Department of Respiratory and Critical Care Medicine, Shenzhen Institute of Respiratory Diseases, Shenzhen People's Hospital, First Affiliated Hospital of Southern Science and Technology University, Second Clinical Medical College of Jinan University, Shenzhen, China

**Keywords:** high-fat diet, metformin, anxiety, gut microbiota, obesity

## Abstract

The pathogenic factors of the complex epidemic disorder–obesity, have expanded from genetic background, endocrine factors, abnormal feeding behaviors, and direct neural control of adipose tissue physiology. As a chronic metabolic disease, it is important to find new potential therapeutic targets and locate a sensitive time window for intervention. In this study, we focus on the early stage of a high-fat diet mouse model: a short-term 3-week treatment. Our results showed that this short-term 3-week HFD can already induce significant body weight gain, increased adipocyte size and surprisingly, anxiety-like behavior of the animals. Then we tried the early intervention with metformin, already reported for its effects in long-term HFD induced obesity. For a short-term 3-week co-treatment, metformin alleviated the HFD-induced increase in body weight, the increase in adipocyte size and furthermore, the anxiety-like behavior. Differences were noted among the normal diet (ND), HFD, and HFD with metformin co-treatment groups in gut microbiota, including its composition and diversity. The possible involvement of gut microbiota cannot be ruled out. Intense phospho-AMPK staining was found in the metformin treatment group in the habenular nuclei, hippocampus and basal ganglia of the brain compared with the HFD group, implying that the anxiolytic effect of metformin could be due to the direct activation of the AMPK pathway in the anxiety-related brain nuclei.

## Introduction

The rising epidemic of obesity calls for increased effort to identify new therapeutic targets/strategies for the treatment of this metabolic disorder. The pathogenic factors of obesity have expanded from genetic background and endocrine factors to central nervous control, including abnormal feeding behaviors and direct neural control of adipose tissue physiology (1, 2). Considerable progress has been made over the past few decades; however, more research is needed to solve the questions surrounding this disorder.

Metformin (dimethylbiguanide) has become a first-line oral blood glucose-lowering agent for patients with type 2 diabetes mellitus (T2DM) (3, 4). In patients with T2DM, who are also obese, metformin also plays a clinical role in obesity (5, 6). The detailed functional mechanism of its action in obesity needs further investigation. Metformin is derived from galegine, a natural product from a medieval European herbal medicine plant *Galega officinalis*. Established as a safe and effective therapy, metformin has multiple modes of actions and its molecular mechanisms are not fully deciphered, despite its clinical usage for over 60 years (3, 4, 7). One of the major molecular targets of metformin is the cellular energy sensor Adenosine monophosphate (AMP)-activated protein kinase (AMPK) (7, 8). Besides hepatic gluconeogenesis, accumulating tissue/organ targets were found for metformin, including white and brown adipose tissue (9), lung (10), and the central nervous system (11).

Although it is known to have a background in genetic and environmental factors, one direct cause of obesity remains an imbalance between caloric intake and expenditure.In this study, a high-fat diet (HFD) mouse model was used. For its chronic characteristics and the complexity of energy expenditure, we focused on the early phase of HFD feeding in relation to body weight gain. Thus, the HFD treatment lasted for 3 weeks. Considering the current implications of the alteration of gut bacteria in both obesity (12) and metformin-treated diabetes (13), the changes in gut microbiota under the short-term HFD and metformin co-treatment was also investigated. In addition, possible central nervous effects of the HFD were evaluated by a free-moving behavioral test in the Elevated plus maze (EPM) and the Open field test (OFT). Considering the reported activation of the AMPK pathway by metformin, the immunostaining of phospho-AMPK was carried out in WAT and the brain.

## Materials and Methods

### Mice

Adult (6 weeks) male C57BL/6J mice (Beijing Vital River Laboratory Animal Technology Co., Ltd. China) were group-housed, given access to food pellets and water *ad libitum*, and maintained on a 12:12-h light/dark cycle. All husbandry and experimental procedures in this study were approved by the Animal Care and Use Committees of the Shenzhen Institute of Advanced Technology (SIAT), Chinese Academy of Sciences (CAS), China.

### High-Fat Diet (HFD), Normal Diet (ND), and Metformin Treatment

Three groups of mice were subjected to different treatments: one group received the normal diet (ND); one group received the high-fat diet (HFD); and a third group received the HFD diet and co-treatment with metformin via oral gavage (300 mg/kg/day, Sigma-Aldrich, BP227, St. Louis, MO). Saline was administered to the ND and HFD groups via oral gavage. For HFD, 60% of the energy was derived from fat, while in the ND, 10% of the energy was derived from fat (Trophic Animal Feed High-Tech, China; TP23300 for HFD, TP23302 for ND). The formula for the HFD was as follows: casein (267 g/kg), maltodextrin (157 g/kg), sucrose (89 g/kg), soybean oil (33 g/kg), lard oil (301 g/kg), cellulose (67 g/kg), mineral mix M1020 (66 g/kg), vitamin mix V1010 (13 g/kg), L-cystine (4 g/kg), choline bitartrate (3 g/kg), TNHQ (0.067 g/kg).

All treatments lasted for 3 weeks. Body weight gain was recorded for each mouse daily until day 21. On the morning of day 21, body weights of mice were recorded, fecal samples were collected and then the mice were subjected to Elevated plus maze test (EPM). On day 22, Open field test (OFT) was performed, then the mice were deep anesthetized and sacrificed. Epididymal white adipose tissues and the brain from three mice of each group were subjected for further histological study. Fecal samples of the mice were collected into sterile tubes, snap-frozen, and then stored at −80°C until the day of analysis.

### Histological Study

While under deep anesthesia, mice were transcardially perfused with 4% paraformaldehyde (PFA) in PBS, epididymal white adipose tissues (WAT) and the brain were harvested and posted-fixed in 4% PFA. The WAT tissue samples were embedded in paraffin, and 4 μm sections were cut on a microtome. The sections were subjected to hematoxylin and eosin (H&E) staining for morphological examination and immune fluorescent staining for phospho-AMPK (pAMPK). For adipocyte size analysis, the longer diameter of each adipocyte was measured. To avoid clustered analysis, the readings from one mouse were first averaged and used its mean as a single value for further comparison between different treatment groups. To evaluate the adipocyte enlargement, the mean of all adipocytes counted were calculated and adipocyte with a larger diameter than the mean of all cells measured in the ND group was defined as “Large adipocyte.” The number and the percentage of Large adipocyte were calculated as a parameter of adipocyte enlargement.

Also, brains harvested were post-fixed with 4% PFA, cryoprotected in 30% sucrose in PBS and cut on a cryostat in 30 μm slices. Immunohistochemistry was performed to map the pAMPK expression in the brain. Antibody staining was performed on single-well floating tissue sections. Sections were incubated for 24 h in primary antibodies at 4°C followed by overnight incubation with secondary antibodies at 4°C. The primary antibody used was rabbit anti-pAMPK (#2535, Cell Signaling Technology;1:50). Suitable secondary antibodies were chosen to reveal different fluorescent colors. For counterstaining, sections were incubated for 10 min with 40, 6-diamidin-2-phenylindol (DAPI, 0.4 mg/mL, Sigma). All the images were captured with a Zesis LSM 880 confocal microscope or an Olympus VS120 virtual microscopy slide scanning system.

### DNA Extraction, 16S Ribosome RNA V4 Region Sequencing and Analysis

DNA extraction was carried out according to the manufacturer's instructions–MOBIO PowerSoil® DNA Isolation Kit 12888-100. DNA was stored at −80°C in Tris-EDTA buffer solution. To enable amplification of the V4 region of the 16S rRNA gene and add barcode sequences, unique fusion primers were designed based on the universal primer set, 515F (5′-GTGYCAGCMGCCGCGGTAA-3′) and 806R (5′-GGACTACNVGGGTWTCTAAT-3′), along with barcode sequences. PCR mixtures contained 1 μL of each forward and reverse primers (10 μM), 1 μL of template DNA, 4 μL of dNTPs (2.5 mM), 5 μL of 10 × EasyPfu Buffer, 1 μL of Easy Pfu DNA Polymerase (2.5 U/μL), and 1 μL of double-distilled water in a total 50 μL reaction volume. Thermal cycling consisted of an initial denaturation step at 95°C for 5 min, followed by 30 cycles of denaturation at 94°C for 30 s, annealing at 60°C for 30 s, and extension at 72°C for 40 s, with a final extension step at 72°C for 4 min. The expected band size for 515f-806r is ~300–350 bp checked by agarose gel. Quantify amplicons with Quant-iT PicoGreen dsDNA Assay Kit (ThermoFisher/Invitrogen cat. no. P11496). The amplicon library for high-throughput sequencing on the Illumina MiSeq platform by Promegene, China was combined an equal amount and subsequently quantified (KAPA Library Quantification Kit KK4824) according to manufacturer's instructions. Using the Quantitative Insights into Microbial Ecology (QIIME) 1.8.0 pipeline 1, the raw sequences were processed to concatenate reads into tags according to the overlapping relationship, then, reads belonging to each sample were separated with barcodes and low-quality reads were removed. The processed tags were clustered into the operational taxonomic units (OTUs) at the commonly used 97% similarity threshold. The OTUs were assigned to taxa by matching to the Greengenes database (Release 13.8). A phylogenetic tree of representative sequences was built. Alpha and beta diversity analyses were performed. Distances were calculated with R (3.3.1, flexmix package).

### Elevated Plus Maze (EPM) and Behavioral Analysis

Mice were placed on a four-arm plus maze with two open arms and two closed arms (white PVC, 30 cm in length per arm × 5 cm in width), which was raised 50 cm above the ground for a 15 min session. The EPM was cleaned between mice with 20% ethanol solution. The number of entries to the open arms, time spent in the open arms, and distance traveled in the open arms were recorded and analyzed by Anymaze® software (Stoelting Co., IL, USA).

### Open Field Test (OFT) and Behavioral Analysis

An open field arena (50 cm × 50 cm × 50 cm) made of white PVC was used to assess both locomotor activity and anxiety-like behavior of the animals. The entries to the Center, the time in the Center, the distance traveled in the Center, total distance traveled in the OF, average speed in the OF for a 5 min session were recorded and then analyzed by the Anymaze®software (Stoelting). The open field was cleaned between mice with 20% ethanol solution.

### Statistical Analysis

**Figure 1:**
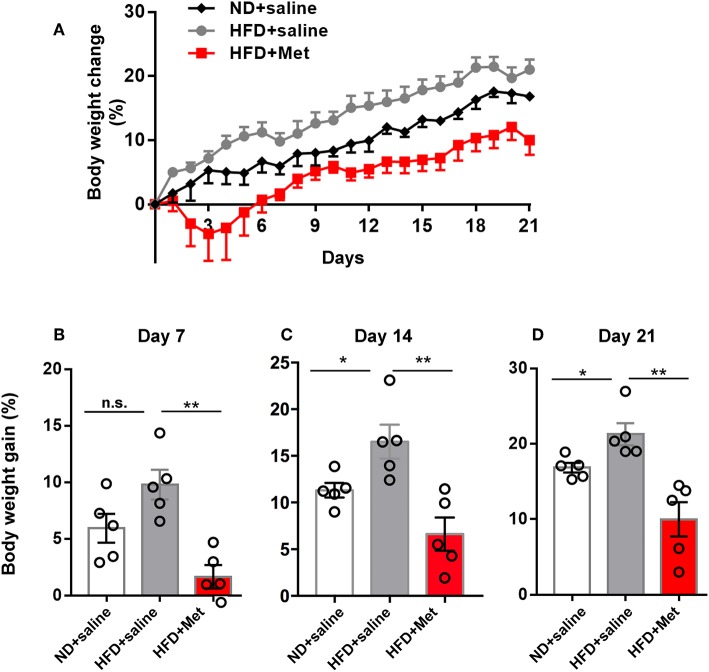
High-Fat Diet (HFD) for 3 weeks induced significant body weight gain compared with Normal Diet (ND) group, which was rescued by metformin co-treatment. **(A)** Three groups of mice were subjected to different treatments, one group for normal diet (ND + saline), one group for high fat diet (HFD + saline) and a third group for HFD diet with oral garage co-treatment of metformin (HFD + Met), the gain of body weight as percentage of original body weight were demonstrated; **(B)** on day 7, HFD + Met group showed a low body weight gain compared with HFD group; **(C,D)** on day 14 and day 21, HFD group showed higher body weight gain compared with ND, which was rescued by metformin co-treatment. For **(A–D)**, data were presented as mean ± SEM, *n* = 5 per group, each dot represents one mouse; One-way ANOVA with Tukey's test, ^*^p < 0.05, ^**^p < 0.01.

**Figure 2:**
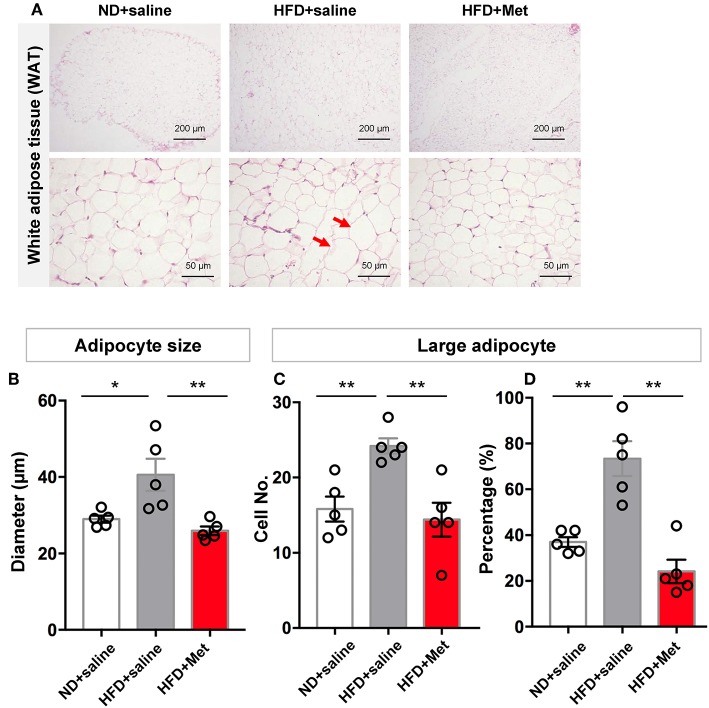
Three-week HFD induced morphological change in white adipose tissue (WAT) and adipocyte size increase, which were alleviated by metformin co-treatment. **(A)** Representative H&E staining figures show that the changes in gain of body weight was accompanied by morphological change in the adipocytes of WAT (arrow heads indicate enlarged adipocytes; scale bars, 200 and 50 μm, respectively); **(B)** HFD group had a larger averaged adipocyte size compared with ND, which was rescued by metformin co-treatment; **(C,D)** for large adipocytes (defined as cells with a diameter larger than the mean of all cells in ND group), HFD group showed higher value in cell number and percentage of large adipocytes. This increase was also rescued by metformin co-treatment. For **(A–D)**, *n* = 5 per group; for **(B–D)**, data were presented as mean ± SEM, each dot represents one mouse; One-way ANOVA with Tukey's test, *p < 0.05, **p < 0.01.

**Figure 3:**
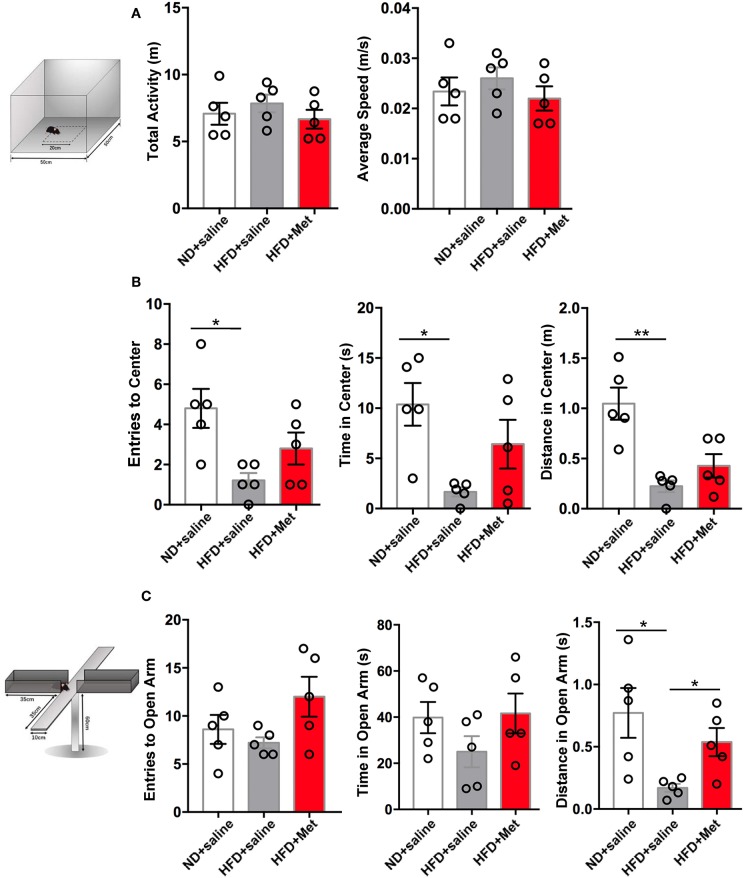
Three-week HFD induced anxiety-like behavior, which were alleviated by metformin co-treatment. **(A)** The total activity and average speed of the animals were not affected by HFD or HFD + Met in the open field; **(B)** in the Open field test (OFT), Entries to Center, Time in Center, and Distance in Center decreased in HFD group compared with ND group; the difference between HFD vs. HFD + Met was not significant; **(C)** in the Elevated plus maze test (EPM), Distance in Open arm decreased in HFD compared with ND, which was alleviated by metformin co-treatment. For **(A–C)**, data were presented as mean ± SEM, *n* = 5 per group, each dot represents one mouse; One-way ANOVA with Tukey's test, *p < 0.05, **p < 0.01.

Data were expressed as mean ± SEM (Figures 1–**3**) and Box and Whiskers (**Figure 5**). Statistical significance was set at *p* < 0.05 (^*^p < 0.05, ^**^p < 0.01, ^****^p < 0.0001) and power analysis (Cohen's *d*) was carried out. All *n* values represent the number of mice used in each experiment. One-way analysis of variance (ANOVA) with Tukey's multiple comparisons test or permutation test was used as appropriate.

## Results

### Three-Week High-Fat Diet (HFD) Induced Significant Body Weight Gain and an Increase in the Adipocyte Size, Which Were Alleviated by Metformin Co-treatment

Three groups of mice were subjected to different treatments: one group to the normal diet (ND + saline); one group to the high-fat diet (HFD + saline); and a third group to the HFD diet and co-treatment with metformin (HFD + Met) via oral gavage. All treatments lasted 3 weeks. Body weight gain was recorded daily until day 21 (Figure 1A). The average body weight of animals in each group showed no significant differences before treatment on day 0 (20.20 ± 0.45 g of the ND group, 20.59 ± 0.36 g of HFD group, 21.31 ± 0.39 g for HFD + Met group; *n* = 5 for each group, One-way ANOVA with Tukey's test, ND vs. HFD, *p* = 0.776, Cohen's *d* = 0.48; HFD vs. HFD + Met, *p* = 0.429, Cohen's *d* = 1.25). At the end of 3 weeks, mice in the HFD group showed an increase in body weight gain, compared to the ND group, whereas this increase was alleviated by metformin co-treatment (Figures 1B–D, *n* = 5 for each group, each dot represents one animal, data presented as body weight gain/original body weight; Figure 1B, ND vs. HFD, *p* > 0.05, Cohen's *d* = 0.43; HFD vs. HFD + Met, *p* < 0.01, Cohen's *d* = 3.09; Figure 1C, ND vs. HFD, *p* < 0.05, Cohen's *d* = 1.85; HFD vs. HFD + Met, *p* < 0.01, Cohen's *d* = 2.75; Figure 1D, ND vs. HFD, *p* < 0.05, Cohen's *d* = 1.94; HFD vs. HFD + Met, *p* < 0.01, Cohen's *d* = 2.93). Besides, the difference between HFD and HFD + Met was significant already as early as day 7, suggesting the early onset of metformin's effect on body weight gain.

Representative images of epididymal white adipose tissue (WAT) stained with H&E (Figure 2A) showed that the changes in body weight gain were accompanied by morphological changes in the adipocytes, with enlarged adipocytes in the HFD group, compared to the ND group and HFD + Met group. The longer diameter of each adipocyte was measured to quantify the change in adipocyte size. The result showed that HFD group had a larger averaged adipocyte size compared with ND, which was rescued by metformin co-treatment (Figure 2B, *n* = 5 for each group, each dot represents one animal, for ND vs. HFD, *p* < 0.05, Cohen's *d* = 1.90, for HFD vs. HFD + Met, *p* < 0.01, Cohen's *d* = 2.38). Defining “Large adipocyte” as cells with a diameter larger than the mean of all cell measured in ND group, we found that HFD group had more Large adipocyte cell number and cell percentage than ND, which were also rescued by metformin co-treatment (*n* = 5 for each group, each dot represents one animal, Figure 2C, for ND vs. HFD, *p* < 0.01, Cohen's *d* = 3.05, for HFD vs. HFD + Met, *p* < 0.01, Cohen's *d* = 2.81; Figure 2D, for ND vs. HFD, *p* < 0.01, Cohen's *d* = 3.26, for HFD vs. HFD + Met, *p* < 0.01 Cohen's *d* = 3.79). These data suggest that metformin co-treatment had a suppressive effect on HFD-induced body weight gain and adipocyte enlargement.

### Three-Week HFD Induced Anxiety-Like Behaviors, Which Were Alleviated by Metformin Co-treatment; Metformin Increased pAMPK Levels in the WAT and in the Habenular Nuclei, Hippocampus and Basal Ganglia of the Brain

Considering clinical reports about the interaction between obesity and anxiety (14), we further evaluated the possible variance in anxiety-like behavior in OPT and EPM. In OPT, the entries to the Center, the time in the Center and the distance in the Center all indicated that HFD induced anxiety-like behaviors. There was a trend for metformin to rescue this effect, but not significant (Figure 3B, *n* = 5 for each group, each dot represents one animal; for Entries to Center, ND vs. HFD, *p* < 0.05, Cohen's *d* = 2.45, HFD vs. HFD + Met, *p* > 0.05, Cohen's *d* = 0.83; for Time in Center, ND vs. HFD, *p* < 0.05, Cohen's *d* = 2.84, HFD vs. HFD + Met, *p* > 0.05, Cohen's *d* = 1.36; for Distance in Center, ND vs. HFD, *p* < 0.01, Cohen's *d* = 3.43, HFD vs. HFD + Met, *p* > 0.05, Cohen's *d* = 1.11). In EPM, the HFD showed its effect in inducing anxiety like behavior and rescued by the metformin co-treatment. The distance traveled in the open arm decreased in HFD compared with ND, which was alleviated by metformin co-treatment (Figure 3C, *n* = 5 for each group, each dot represents one animal; ND vs. HFD, *p* < 0.05, Cohen's *d* = 2.10, HFD vs. HFD + Met, *p* < 0.05, Cohen's *d* = 2.21). The locomotor of the animal was not affected by the 3-week HFD treatment or HFD-Met treatment (Figure 3A, *n* = 5 for each group, each dot represents one animal; for total activity, *ps* > 0.05, Cohen's *ds* = 0.52 and 0.86; for average speed, *ps* > 0.05, Cohen's *ds* = 0.53 and 0.87). Though there was no correlation between the body weight changes on day 21 and the parameters of OFT and EPM tests (all R squared < 0.26, all *ps* > 0.05), while the parameters of OFT and EPM tests were strongly correlated (for Distance in Open Arms of EPM vs. Distance in Center of OFT, R squared = 0.43, *p* < 0.01).

**Figure 4:**
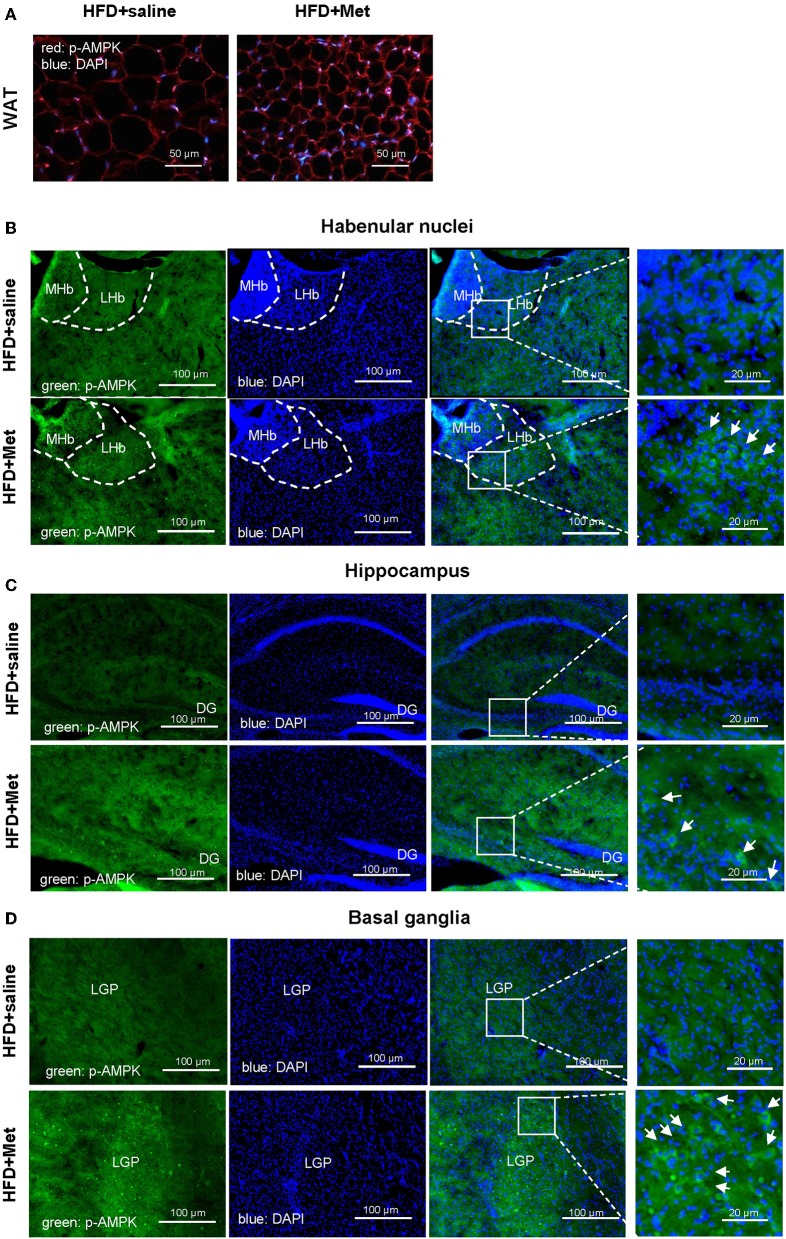
Metformin co-treatments showed stronger pAMPK immunostaining in the WAT and in the habenular nuclei, hippocampus and basal ganglia of the brain. Compared with HFD group, HFD + Met group showed stronger pAMPK staining in the adipocytes (**A**; red, pAMPK, blue, DAPI; scale bars, 50 μm) and in the habenular nuclei, hippocampus and basal ganglia [for **(B–D)**, green, pAMPK, blue, DAPI, scale bars, 100 and 20 μm, respectively; MHb, medial habenula nucleus; LHb, lateral habenular nucleus; DG, dentate gyrus; LGP, lateral globus pallidus; for **(A–D)**, *n* = 3 per group].

We next want to know whether the anxiolytic effect of metformin could be partially due to its direct action in the brain. As metformin is a well-known AMPK activator and has been reported for its central function in the brain (15) and AMPK is a primary sensor of cellular energy states and regulates cellular energy metabolism, we examined the possible activation of AMPK pathway by metformin treatment. Stronger pAMPK immunostaining was found in the metformin co-treatment group, not only in the WAT (Figure 4A), but also in the brain, located in the habenular nuclei, hippocampus and basal ganglia, compared with the HFD group (Figures 4B–D). Habenular nuclei, especially lateral habenular nucleus (LHb), hippocampus, and basal ganglia are all involved with anxiety-related disorders (16–21). Our data suggested that the anxiolytic effect of metformin co-treatment could be due to the direct activation of the AMPK pathway in the anxiety-related brain nuclei.

### The HFD and HFD + Metformin Treatments Changed Microbiota Diversity and Altered Its Composition

**Figure 5:**
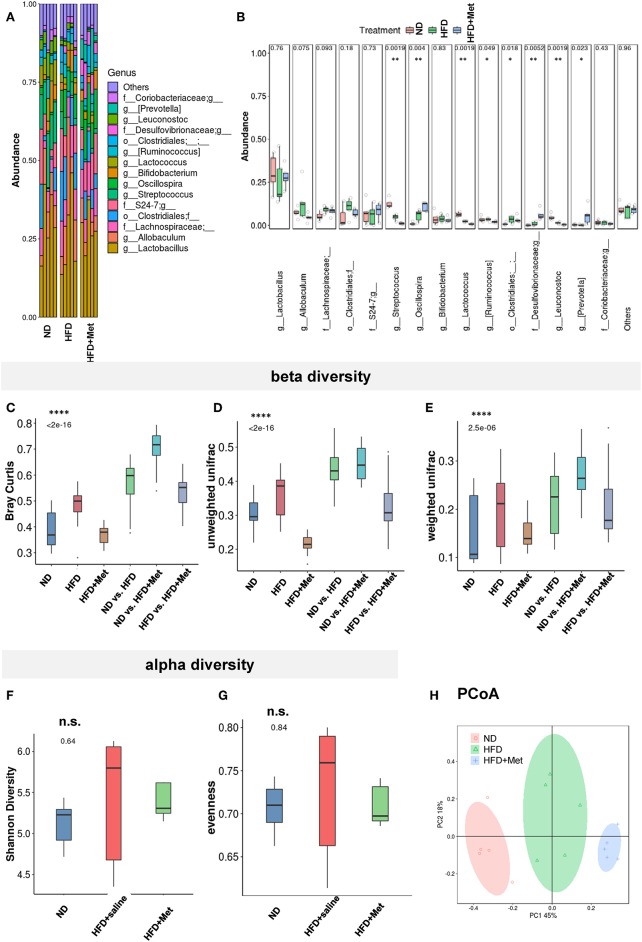
The HFD and HFD + metformin treatments changed microbiota diversity and altered its composition. **(A,B)** The abundance of species varied among ND, HFD, and HFD + Met group with significant difference for *Streptococcus, Oscillospira, Ruminococcus, Leuconostoc*, and *Prevotella*; **(C–E)** beta diversity analysis showed significant difference between groups after Bray-curtis dissimilarity, unweighted UniFracand weighted UniFrac analysis (****p < 0.0001); **(F,G)** no significant difference was shown by alpha diversity analysis of Shannon's index or evenness; **(H)** Principal Co-ordinates Analysis (PCoA) revealed the difference in gut bacteria composition in the three distinct groups. For **(A-H)**, n = 5 for each group, box and whiskers was plotted; One-way ANOVA with permutation test, *p < 0.05, **p < 0.01, ****p < 0.0001, n.s. not significant.

The close association between altered gut microbiota and obesity (12) or long-term high-fat diet (22, 23) has been established. Considering the significant effect of metformin in rescuing the 3-week HFD-induced body weight gain, we accessed the impact of HFD and HFD + Met on the gut microbiota through 16S rRNA gene sequencing of the fecal contents of the animals (Figures 5A–H, *n* = 5 for each group). The bacterial abundance of each group varied at genus levels (Figure 5A). With *Firmicutes* and *Bacteroidetes* dominant at phylum level, the top dominant species included *Lactobacillus, Allobaculum, Streptococcus, Oscillospira, Bifidobacterium, Lactococcus, Ruminococcus, Leuconostoc, Prevotella*, among which *Streptococcus, Oscillospira, Ruminococcus, Leuconostoc* and *Prevotella* showed significant difference between groups (Figure 5B, all Cohen's *d*s > 0.8). Beta diversity analysis showed significant difference between groups after Bray-curtis dissimilarity (Figure 5C), unweighted UniFrac (Figure 5D) and weighted UniFrac analysis (Figure 5E, *p* < 0.0001 for all tests), though no significant difference was shown by alpha diversity analysis, either by evenness or Shannon's index (Figures 5F,G). The difference in gut bacteria composition was revealed by Principal Co-ordinates Analysis (PCoA) plotted into three distinct groups (Figure 5H). Though no species was identified by LEfSe with LDA larger than 2, when comparison was made at species level, the *Enterococcus, Leuconostoc, Lactococcus, Streptococcus, Blautia, Coprococcus, Oscillospira, Anaerovorax*, and *Flexispira* were significant different between ND and HFD groups (Supplementary Table 1A, ND vs. HFD, all *ps* < 0.05). Among all the above species modified by HFD, the *Enterococcus, Lactococcus, Streptococcus* demonstrated difference after metformin co-treatment (Supplementary Table 1B, HFD vs. HFD + Met, all *ps* < 0.05). The presence of *Akkermansia* was reported to inversely correlates with body weight in rodents and humans (24). Interestingly, we found that though the level of Species *Akkermansia* was not influenced by HFD diet compared with ND (ND vs. HFD, *p* = 0.438, Cohen's *d* = 0.27), it was different after metformin treatment (HFD vs. HFD + Met, *p* < 0.05, Cohen's *d* = 0.34).

## Discussion

Obesity is associated with an increased risk of metabolic disorders and cardiovascular diseases, as well as emotional disorders (14, 25–28). In addition, mice fed on HFD displayed learning and memory impairment (29), as well as emotional related disorders, such as depressive-like (30–35) and anxiety-like behavior (36–38). However, the previous all focused on the related effects after long-term HFD treatment, such as 12–18 weeks (30, 31, 33–37, 39, 40), or even 24 weeks (38). As a chronic metabolic disease, it is important to find new potential therapeutic targets and locate a sensitive time window for intervention. In this study, we focus on the early stage of an HFD model: a short-term 3-week treatment. We found that even short-term 3-week-HFD treatment caused significant body weight gain, increased adipocyte size and induced anxiety-like behavior in the animals. This is indicative of a quicker onset of the effects of dietary change on emotional states, which had not been revealed by previous studies. The anti-obesity role of metformin in the HFD model usually adopt a longer treatment period too, for example, 10-week metformin treatment in a 28-week HFD-induced obesity model (41). In our study, the rescue effect of metformin was proved at 3-week period, evident already on day 7 of co-treatment, suggesting the early on-set of the beneficial drug effect.

It is also exciting to find out that 3-week metformin co-treatment can already alleviated not only the metabolic body weight gain effect of HFD, but also the emotional aspect of anxiety-like behavior. Actually, the central roles of metformin have been reported, which includes the promotion of neurogenesis through the atypical protein kinase C-CREB-binding protein (PKC-CBP) pathway (15), learning and memory improvement in association with glucagon-like peptide-1 (42) and AMPK dependent autophagic pathway in an ischemia model (34). Current results imply that the model adopted in the present study could be a candidate animal model for further study of the central effects of metformin in diet-induced anxiety disorders. In our study, we found that pAMPK levels in anxiety-related brain regions like LHb (16, 17), hippocampus (18, 19), and basal ganglia (20, 21) were increased compared with the HFD group. This anxiolytic effect of metformin through the AMPK pathway was in line with a previous model of transient forebrain ischemia model (34).

The association between gut microbiota and obesity has been intensively studied in both clinical and animal studies (12, 24, 43). The effects of metformin in improving T2DM (13) and obesity (41) have been proposed to be partially mediated by modifications in the gut microbiota. Here, we showed that the short-term HFD-induced body weight gain is associated with a shift in the composition of microbiota and increased anxiety. Considering the emerging evidence of the connection between gut microbiota and mental disorders, including autism (44–46), depression (47–49), and anxiety disorders (50, 51), and the impact of HFD and HFD-Met on the anxiety-like behavior of the animals, we also studied the changes in gut microbiota under the short-term HFD and metformin co-treatment. Our data showed that differences were noted among the normal diet (ND), HFD, and HFD with metformin co-treatment groups in gut microbiota, including its composition and diversity. We found that the level of *Akkermansia* increased after metformin treatment in our study, which was in line with previous reports (52, 53). A recent study demonstrated beneficial outcomes from the administration of *Akkermansia* in overweight/obese insulin-resistant volunteers (54). Considering that, it is intriguing to study the detailed action and pathways of *Akkermansia* in the rescue of body weight gain of HFD by metformin, especially at the early stage of the process in the future study.

The beneficial effect of metformin is evident at an early stage of HFD-induced obesity development in aspects of white adipose tissue cellular morphology, the anxiety level of the animals. Also, the possible involvement of gut microbiota cannot be ruled out. And the anxiolytic effect of metformin co-treatment could be due to the direct activation of the AMPK pathway in the anxiety-related brain nuclei. Deciphering further details regarding the gut-brain-axis would foster a better understanding of the mechanisms associated with HFD and anxiety-like behaviors.

## Ethics Statement

Adult (6 weeks) male C57BL/6J mice (Beijing Vital River Laboratory Animal Technology Co., Ltd. China) were group-housed, given access to food pellets and water *ad libitum*, and maintained on a 12:12-h light/dark cycle. All husbandry and experimental procedures in this study were approved by Animal Care and Use Committees at the Shenzhen Institute of Advanced Technology (SIAT), Chinese Academy of Sciences (CAS), China.

## Author Contributions

LL designed the study. SJ, LW, and LL performed experiments. LL wrote the manuscript.

### Conflict of Interest

The authors declare that the research was conducted in the absence of any commercial or financial relationships that could be construed as a potential conflict of interest.
