# Supplementary material for: Effect of Metformin on Short-Term High-Fat Diet-Induced Weight Gain and Anxiety-Like Behavior and the Gut Microbiota
Source: Front Endocrinol (Lausanne). 2019 Oct 18;10:704. doi: 10.3389/fendo.2019.00704 (PMC6813541; doi:10.3389/fendo.2019.00704)
Supplement: Supplementary file 1 [file Table_1.DOCX]

**Supplementary Table 1. Gut microbiota difference between ND vs. HFD (1.A) and HFD vs. HFD+Met (1.B).**

**1.A.**

| **ID of gut microbiota (ND vs. HFD) #** | **ND_mean** | **HFD_mean** | **t adjust** | **perm pvalue (p<0.05)** |
| --- | --- | --- | --- | --- |
| k__Bacteria;p__Actinobacteria;c__Actinobacteria;o__Bifidobacteriales;f__Bifidobacteriaceae;g__ | 0.0085 | 0.0028 | 0.2260 | 0.0083 |
| k__Bacteria;p__Firmicutes;c__Bacilli;o__Lactobacillales;__;__ | 0.0030 | 0.0006 | 0.2451 | 0.0159 |
| k__Bacteria;p__Firmicutes;c__Bacilli;o__Lactobacillales;f__Enterococcaceae;g__Enterococcus | 0.0040 | 0.0012 | 0.0203 | 0.0079 |
| k__Bacteria;p__Firmicutes;c__Bacilli;o__Lactobacillales;f__Lactobacillaceae;__ | 0.0046 | 0.0014 | 0.2451 | 0.0253 |
| k__Bacteria;p__Firmicutes;c__Bacilli;o__Lactobacillales;f__Leuconostocaceae;g__Leuconostoc | 0.0475 | 0.0137 | 0.1572 | 0.0076 |
| k__Bacteria;p__Firmicutes;c__Bacilli;o__Lactobacillales;f__Streptococcaceae;g__Lactococcus | 0.0647 | 0.0224 | 0.0203 | 0.0084 |
| k__Bacteria;p__Firmicutes;c__Bacilli;o__Lactobacillales;f__Streptococcaceae;g__Streptococcus | 0.1257 | 0.0482 | 0.0345 | 0.0082 |
| k__Bacteria;p__Firmicutes;c__Clostridia;o__Clostridiales;__;__ | 0.0090 | 0.0333 | 0.2451 | 0.0481 |
| k__Bacteria;p__Firmicutes;c__Clostridia;o__Clostridiales;f__Christensenellaceae;g__ | 0.0013 | 0.0004 | 0.2354 | 0.0230 |
| k__Bacteria;p__Firmicutes;c__Clostridia;o__Clostridiales;f__Lachnospiraceae;__ | 0.0530 | 0.0918 | 0.2354 | 0.0379 |
| k__Bacteria;p__Firmicutes;c__Clostridia;o__Clostridiales;f__Lachnospiraceae;g__Blautia | 0.0007 | 0.0000 | 0.1011 | 0.0082 |
| k__Bacteria;p__Firmicutes;c__Clostridia;o__Clostridiales;f__Lachnospiraceae;g__Coprococcus | 0.0007 | 0.0087 | 0.2354 | 0.0159 |
| k__Bacteria;p__Firmicutes;c__Clostridia;o__Clostridiales;f__Peptococcaceae;g__ | 0.0000 | 0.0013 | 0.2451 | 0.0087 |
| k__Bacteria;p__Firmicutes;c__Clostridia;o__Clostridiales;f__Ruminococcaceae;g__Oscillospira | 0.0108 | 0.0561 | 0.2354 | 0.0175 |
| k__Bacteria;p__Firmicutes;c__Clostridia;o__Clostridiales;f__[Mogibacteriaceae];g__Anaerovorax | 0.0001 | 0.0007 | 0.2983 | 0.0251 |
| k__Bacteria;p__Firmicutes;c__Erysipelotrichi;o__Erysipelotrichales;f__Erysipelotrichaceae;__ | 0.0078 | 0.0002 | 0.2354 | 0.0075 |
| k__Bacteria;p__Proteobacteria;c__Deltaproteobacteria;o__Desulfovibrionales;f__Desulfovibrionaceae;g__ | 0.0007 | 0.0097 | 0.3487 | 0.0464 |
| k__Bacteria;p__Proteobacteria;c__Epsilonproteobacteria;o__Campylobacterales;f__Helicobacteraceae;g__Flexispira | 0.0000 | 0.0071 | 0.3679 | 0.0492 |
| k__Bacteria;p__Tenericutes;c__Mollicutes;o__RF39;f__;g__ | 0.0017 | 0.0000 | 0.2983 | 0.0251 |

***^#^*** *When comparison was made at species level between ND and HFD, IDs of gut microbiota with perm p value<0.05 were listed.*

**1.B**

| **ID of gut microbiota* (HFD vs. HFD+Met) ##** | **HFD_mean** | **HFD+Met_mean** | **t adjust** | **perm pvalue (p<0.05)** |
| --- | --- | --- | --- | --- |
| k__Bacteria;p__Actinobacteria;c__Actinobacteria;o__Bifidobacteriales;f__Bifidobacteriaceae;g__ | 0.0028 | 0.0005 | 0.3109 | 0.0140 |
| k__Bacteria;p__Bacteroidetes;c__Bacteroidia;o__Bacteroidales;f__Porphyromonadaceae;g__Parabacteroides | 0.0001 | 0.0019 | 0.4771 | 0.0152 |
| k__Bacteria;p__Bacteroidetes;c__Bacteroidia;o__Bacteroidales;f__[Paraprevotellaceae];g__[Prevotella] | 0.0023 | 0.0572 | 0.3773 | 0.0155 |
| k__Bacteria;p__Firmicutes;c__Bacilli;o__Lactobacillales;f__Enterococcaceae;g__Enterococcus | 0.0012 | 0.0001 | 0.3239 | 0.0493 |
| k__Bacteria;p__Firmicutes;c__Bacilli;o__Lactobacillales;f__Leuconostocaceae;g__Leuconostoc | 0.0137 | 0.0040 | 0.2745 | 0.0068 |
| k__Bacteria;p__Firmicutes;c__Bacilli;o__Lactobacillales;f__Streptococcaceae;g__Lactococcus | 0.0224 | 0.0073 | 0.0255 | 0.0083 |
| k__Bacteria;p__Firmicutes;c__Bacilli;o__Lactobacillales;f__Streptococcaceae;g__Streptococcus | 0.0482 | 0.0112 | 0.2632 | 0.0071 |
| k__Bacteria;p__Firmicutes;c__Clostridia;o__Clostridiales;f__Clostridiaceae;g__Clostridium | 0.0049 | 0.0002 | 0.4892 | 0.0484 |
| k__Bacteria;p__Firmicutes;c__Clostridia;o__Clostridiales;f__Lachnospiraceae;g__[Ruminococcus] | 0.0326 | 0.0200 | 0.3109 | 0.0318 |
| k__Bacteria;p__Firmicutes;c__Clostridia;o__Clostridiales;f__Peptococcaceae;g__ | 0.0013 | 0.0002 | 0.3773 | 0.0381 |
| k__Bacteria;p__Firmicutes;c__Clostridia;o__Clostridiales;f__Ruminococcaceae;g__Oscillospira | 0.0561 | 0.1098 | 0.2892 | 0.0295 |
| k__Bacteria;p__Firmicutes;c__Erysipelotrichi;o__Erysipelotrichales;f__Erysipelotrichaceae;__ | 0.0002 | 0.0042 | 0.3773 | 0.0149 |
| k__Bacteria;p__Firmicutes;c__Erysipelotrichi;o__Erysipelotrichales;f__Erysipelotrichaceae;g__Allobaculum | 0.1348 | 0.0450 | 0.4892 | 0.0497 |
| k__Bacteria;p__Proteobacteria;c__Deltaproteobacteria;o__Desulfovibrionales;f__Desulfovibrionaceae;g__ | 0.0097 | 0.0615 | 0.2770 | 0.0104 |
| k__Bacteria;p__Verrucomicrobia;c__Verrucomicrobiae;o__Verrucomicrobiales;f__Verrucomicrobiaceae;g__Akkermansia | 0.0001 | 0.0128 | 0.4892 | 0.0470 |

***^##^****When comparison was made at species level between HFD and HFD+metformin, IDs of gut microbiota with perm p value<0.05 were listed.*
